# Advanced Blood–Brain Barrier Drug Delivery

**DOI:** 10.3390/pharmaceutics15010093

**Published:** 2022-12-27

**Authors:** William M. Pardridge

**Affiliations:** Department of Medicine, University of California, Los Angeles (UCLA), Los Angeles, CA 90095, USA; wpardrid@ucla.edu

This Special Issue of Pharmaceutics, “Advanced Blood–Brain Barrier Drug Delivery,” comprises 16 articles or reviews, which cover a cross-section of brain drug delivery for either small-molecule or large-molecule therapeutics. The areas covered include (i) receptor-mediated transport (RMT); (ii) carrier-mediated transport (CMT); (iii) active efflux transport (AET); (iv) lipid nanoparticles (LNP); and (v) in vivo methods for the measurement of blood–brain barrier (BBB) drug transport. Many areas of brain drug delivery are not covered in a designated article but are reviewed in this issue [1]. These areas include drug delivery into the cerebrospinal fluid (CSF) via an intrathecal injection into either the lumbar or ventricular CSF; trans-nasal drug delivery; intra-cerebral brain drug delivery with either intra-cerebral implants or convection enhanced diffusion; BBB disruption via the intra-carotid arterial infusion of noxious agents or the intravenous injection of micro-bubbles in association with focused ultrasound; exosomes; stem cells; or the lipidization of polar small molecules. These latter brain drug delivery technologies have specific limitations, which, to date, have prevented the scalable translation to human neurotherapeutics, as reviewed in this volume [1].

The RMT of biologics across the BBB requires attachment of the biologic to a peptide or peptidomimetic monoclonal antibody (MAb) that binds to a specific endogenous receptor expressed on the luminal membrane of the brain capillary endothelium that forms the BBB. The targeted receptor normally serves to mediate the transport of an endogenous peptide from blood to brain, such as the insulin receptor (IR), transferrin receptor 1 (TfR1), insulin-like growth factor (IGF) 1 receptor (IGF1R), or leptin receptor (LEPR) [1]. The peptide or MAb acts as a molecular Trojan horse to ferry the biologic agent across the BBB via RMT on the endogenous peptide receptor at the brain endothelium. This Special Issue includes seven articles that describe the use of MAb-based Trojan horses targeting the IR, TfR1, IGF1R, or an orphan receptor [2,3,4,5,6,7,8], and one article on the use of peptides as a BBB Trojan horse [9].

The review by Boado [2] describes the genetic engineering of IgG fusion proteins that target either the murine TfR1 for mouse investigations, or the human insulin receptor (HIR) for either brain uptake studies in Old World primates such as the Rhesus monkey, or in human clinical trials. Boado [2] describes the genetic engineering, expression, and validation of TfRMAb and HIRMAb fusion proteins for all four classes of protein biologic drugs: lysosomal enzymes, neurotrophins, decoy receptors, and therapeutic antibodies. In the case of the BBB delivery of a therapeutic antibody, the transporter antibody and the therapeutic antibody are combined to produce a bispecific antibody (BSA) [2]. The first clinical trial of a BBB Trojan horse was reported in 2018, which described the 52-week treatment of children with MPSI with weekly intravenous (IV) infusions of a fusion protein of the HIRMAb and the lysosomal enzyme that is mutated in MPSI, α-L-iduronidase (IDUA), and this HIRMAb-IDUA fusion protein is designated valanafusp alfa [2].

The review by Sonoda et al. [3] describes the genetic engineering of a fusion protein, designated pabinafusp alfa, which is formed by a fusion of the mature human iduronate 2-sulfatase (IDS) lysosomal enzyme to the carboxyl terminus of the heavy chain (HC) of a TfRMAb specific for the human TfR1. IDS is mutated in MPS II (Hunter syndrome), where the accumulation of glycosaminoglycans (GAG) in the brain leads to cognitive impairment early in the life of subjects with mutations in the human IDS gene. Pabinafusp alfa treatment of humans with MPSII results in a decrease in GAG levels in CSF [3]. Pabinafusp alfa received regulatory approval in Japan in 2021 [3] and is the first Trojan horse fusion protein to be granted market approval for the treatment of a human disease of the brain.

Decoy receptor therapeutics, such as etanercept, are engineered by fusion of the carboxyl terminus of a soluble extracellular domain (ECD) of a membrane receptor to the amino terminus of a human IgG Fc. Etanercept binds, sequesters, and suppresses the action of the pro-inflammatory cytokine, TNFα. TNFα plays a pro-inflammatory role in AD, but etanercept cannot be used to treat AD because it does not cross the BBB [2]. However, the human TNFR ECD can be re-engineered for BBB penetration and for the treatment of AD with the genetic engineering and application of an IgG-TNFR fusion protein [4]. Ou et al. [4] describe the chronic treatment of 1-year-old double transgenic APP/PS1 AD mice with 2–3 mg/kg of either etanercept or the TfRMAb-TNFR fusion protein administered by intra-peritoneal injection three times a week for 10 weeks. The TfRMAb-TNFR fusion protein, but not etanercept, reduced the Aβ peptide content, thioflavin-S positive Aβ plaques, and insoluble oligomeric Aβ in the brain, in parallel with an increase in Aβ plaque-associated phagocytic microglia [4]. Chronic treatment of the AD mice with the TfRMAb-TNFR fusion protein caused no abnormalities in either hematologic parameters in blood or iron dysregulation in the brain [4].

PD is associated with the deposition in the brain of insoluble aggregates derived from the α-synuclein (SYN) protein, and a MAb that disaggregates SYN plaque is a potential treatment of PD. However, therapeutic antibodies do not cross the BBB [1]. In order to produce a new treatment for PD, a BSA was engineered from the 8D3 mouse TfRMAb and the Syn-02 antibody, as described by Roshanbin et al. [5]. The Syn-02 antibody binds SYN aggregates but not soluble SYN monomers [5]. A single chain Fv (ScFv) form of the 8D3 TfRMAb was fused to the carboxyl terminus of each light chain (LC) of an engineered form of the Syn-02 antibody [5]. L61 transgenic mice that over-express the human SYN protein develop aggregates in the brain by 3 months of age [5]. L61/SYN transgenic mice were treated with 10 mg/kg of either the Syn-2 antibody alone or the 8D3-Syn-02 BSA on days 1, 2, and 4, and then euthanized on day 5. This short course of treatment resulted in a modest decrease in the brain levels of SYN oligomers. Future studies describing a longer duration of treatment are warranted for this novel approach to the reduction in insoluble SYN aggregates in the brain in PD.

The works of Boado [2], Sonoda et al. [3], Ou et al. [4], and Roshanbin et al. [5] developed classical dual-domain antibodies comprised of both an HC and an LC, each with a variable region. In contrast, single-domain antibodies (sdAb)—also called a nanobody owing to their small size of 15 kDa—are formed only by a variable region of the heavy chain (VH). The two sources of sdAbs are sharks, where the shark VH is designated as a variable new antigen receptor (VNAR) antibody, and camelids (e.g., llama), where the camelid VH is designated a VHH. In this Special Issue, the study of Clarke et al. [6] describe the genetic engineering of a BSA comprising a therapeutic antibody and a shark VNAR. Yogi et al. [7] describe the genetic engineering of a fusion protein derived from a camelid VHH and the neuroactive peptide, neurotensin.

Clarke et al. [6] describe the genetic fusion of the 29D7 TrkB agonist antibody and a single-domain shark VNAR antibody against the TfR1, and designated TXB4. The TXB4-TrkB BSA retained high-affinity binding (low nM KD) to both the transporter target, the TfR1, and to the therapeutic target, TrkB [6]. The therapeutic efficacy of the TXB4-TrkB BSA was assessed in a murine 6-hydroxydopamine model of PD. Mice were treated with phosphate buffered saline (PBS) or 2.5–5 mg/kg of the TXB4-TrkB BSA at days −1 and +7 relative to toxin administration. This dose of toxin in the mouse produces a partial lesion, and the number of cells in the substantia nigra immunoreactive with an antibody against tyrosine hydroxylase (TH) was reduced by 27% on the lesioned side compared to the non-lesioned side in the PBS treated mice. However, there was only a 3% reduction in striatal TH on the lesioned side, relative to the contralateral or non-lesioned side, in the PD mice treated with the TXB4-TrkB BSA [6]. Since the BSA was administered 24 h before neurotoxin injection, future work can examine the neuroprotective effects of delayed treatment with the TxB4-TrkB fusion protein following toxin administration.

Yogi et al. [7] describe the isolation of a camelid VHH following llama immunization with the human IGF1R ECD. The optimal VHH was isolated and designated IGF1R5, and humanized following standard protocols [7]. Humanization of the IGF1R5 VHH resulted in several amino acid substitutions across all four of its framework regions (FR), and its humanized form was designated IGF1R5-H2 [7]. This humanization had a significant impact on the affinity of the VHH binding to the IGF1R, which was measured by surface plasmon resonance. The in vivo transport was measured in the rat model for the non-humanized IGF1R5:Fc. The CSF/serum ratio of the antibody was 0.3%, and the brain concentration of the antibody was 11 nM at 24 h following the IV administration of 15 mg/kg of the fusion protein [7]. After the intravenous injection of 5–20 mg/kg of the VHH-Fc fusion protein, a fusion protein of neurotensin and the IGF1R5-human Fc produced a reduction in core body temperature [7].

The work of Boado [2], Sonoda et al. [3], Ou et al. [4], Roshanbin et al. [5], Clarke et al. [6], and Yogi et al. [7] targeted known RMT systems at the BBB, e.g., the IR, TfR1, or IGF1R. To discover orphan RMT systems at the BBB, in this Special Issue, Georgieva et al. [8] describe their work with the 46.1 antibody. This antibody was generated following screening of a human single chain Fv (ScFv) phage library with cultured brain microvascular endothelial cells, which were produced following the retinoic acid differentiation of induced pluripotent stem cells. The lead candidate ScFv antibody, designated 46.1, was isolated and fused to the amino terminus of rabbit IgG Fc [8], which produces a bivalent antibody of ~100 kDa in size. In a pharmacologic application of the 46.1 orphan receptor antibody, Georgieva et al. [8] describe the genetic engineering of a fusion protein of 46.1 ScFv-Fc and mature neurotensin, which is a 13 amino acid (AA) neuropeptide released from a larger precursor. The biologic activity of the fusion protein in vivo was measured by a core body temperature assay and a phencyclidine-induced hyper-locomotor activity assay [8]. The 46.1 ScFv-Fc-neurotensin fusion protein was pharmacologically active in both assays in mice following the IV injection of 20 mg/kg into the retro-orbital vein. The pharmacologic effect in either assay was reduced or not observed following tail vein injection [8].

The articles in this Special Issue used a monoclonal antibody as the BBB molecular Trojan horse to shuttle the fused biologic agent from blood to brain via endogenous RMT systems at the BBB. Peptides that target RMT systems at the BBB can also be used as drug delivery vectors, and Sanchez-Navarro and Giralt [9]—also in this issue—provide a comprehensive review of BBB shuttle peptides. Two classes of peptide are reviewed: synthetic peptides and peptides isolated from phage display [9]. The most widely studied synthetic peptides include (a) peptides with sequences overlapping with AA 130-152 of human apolipoprotein E (apoE), which are low-affinity ligands for the low density lipoprotein receptor (LDLR) or the LDLR-related protein 1 (LRP1); (b) a peptide overlapping with AA 3371-3409 of human apolipoprotein B (apoB), which is a low-affinity ligand for LDLR; (c) angiopep-2, a 19 AA peptide that is a low-affinity ligand for LRP1; and (d) a 29 AA peptide corresponding to a sequence from the rabies virus glycoprotein (RVG), which is a ligand for the nicotinic acetylcholine receptor (nAChR) [9]. However, the immunohistochemical detection of LDLR, LRP1, or the nAChR in the brain shows that these receptors are expressed on brain cells beyond the BBB and not on the brain endothelium in vivo [1]. Therefore, ligands targeting the LDLR, LRP1, or nAChR are unlikely to mediate RMT across the BBB, and alternative transport pathways should be evaluated. Since all of these peptides are strongly cationic with isoelectric points (pI) of 9–10, they may undergo cationic charge-dependent absorptive-mediated transport (AMT) across the BBB [1]. Potential peptide shuttles derived from screening phage peptide libraries are reviewed in this Special Issue [9]. In a typical peptide phage display library, a 15-mer random AA sequence is incorporated in the amino terminus of the bacteriophage P3 minor coat protein [9]. The phage coat protein is a large protein of >400 AA in length, and the activity of the 15 AA sequence that is embedded in the large p3 protein may differ from the shuttle activity of the 15 AA sequence as a free peptide [1].

Small molecules are often assumed to penetrate the BBB owing to the small size of the drug. However, ~98% of all small molecules do not cross the BBB [1]. Small molecule drugs that penetrate the BBB have a MW < 400 Da, form <8 hydrogen bonds with water, and lack an affinity for a BBB active efflux transporter (AET), such as p-glycoprotein. In the past, medicinal chemists have attempted to increase the BBB transport of drugs by blocking polar functional groups on the drug, a process referred to as ‘lipidization.’ However, lipidization of polar drugs by medicinal chemistry rarely leads to new BBB penetrating drugs, since this increases the MW of the drug and renders it unstable in the blood. An alternative approach to the use of medicinal chemistry to enhance the BBB transport of small-molecule drugs is to modify the drug so that it both (a) retains pharmacologic activity, and (b) has a modest to high affinity for transport via one of several CMT systems at the BBB. In this Special Issue, the article by Huttunen et al. [10] is the most comprehensive review, to date, on the use of medicinal chemistry for designing drugs that reach the brain via CMT across the BBB. All of the CMT systems reviewed by the authors [10] are members of the Solute Carrier (SLC) gene family. The problem lies in the complexity of the SLC family of transporters, as there are >400 genes in >60 families of SLC transporters [1]. Therefore, it is crucial to identify which SLC transporter functions at the luminal membrane of the brain capillary endothelium. Based on the available literature data, Huttunen et al. [10] recommend targeting certain SLC transporters, including ASCT1; the alanine-serine-cysteine transporter (SLC1A); the GLUT1 glucose transporter (SLC2A); the CAT1 cationic amino acid transporter, the LAT1 large neutral amino acid transporter (SLC 7A); the MCT1 and MCT8 monocarboxylic acid transporters (SLC16A); the OATP2B1 and OATP1A2 organic anion transporters (SLC21A/SLCO); the OCT1-3 and OCTN1-2 organic anion and organic cation transporters (SLC22A); and the SNAT3 glutamine transporter (SLC38A). In addition, certain vitamins undergo CMT across the BBB via other members of the SLC gene family [1] and are potential targets for medicinal chemists [10].

The SLC transporters at the BBB may be up- or down-regulated in disease, and in this Special Issue, Latif and Kang [11] review the changes in certain SLC transporters at the BBB in motor neuron disease. They review changes in amyotrophic lateral sclerosis for certain SLC transporters, including the ASCT1/2, LAT1, CAT1, MCT1, the carnitine carrier, OCTN2, and the high-affinity choline transporter (CHT1). Regarding BBB choline transport, the more important transporter at the BBB is shown to be the lower affinity choline transporter-like protein 1 (CTL1, SLC44A1) [11].

Not all small-molecule transporters at the BBB may be members of the SLC gene family. In this Special Issue, Kurosawa et al. [12] use new methodology to identify potential candidates for the protein-coupled organic cation (H^+^/OC) transporter. To identify potential candidates for the BBB organic cation transporter, the authors [12] develop a new methodology, the proteomics-based identification of transporters by crosslinking substrate using the keyhole (PICK) method. This new methodology identified the TM7SF3 transmembrane 7 superfamily member 3 and the LHFPL tetraspan subfamily 6 proteins as potential candidates for the H^+^/OC transporter. Human TM7SF3 is a widely expressed glycosylated membrane protein comprising seven transmembrane regions and 570 AA, including a 21 AA signal peptide (NP_057635). Human LHFPL is a membrane protein comprising four transmembrane regions and 236 AA, with no predicted signal peptide sequence, no predicted N-linked glycosylation sites, and an alanine-rich amino terminus (NP_945351).

Certain BBB transporters mediate the transport of endogenous ligands or drugs in the brain-to-blood direction, and they are active efflux transporters (AET). In this Special Issue, Ronaldson and Davis [13] review the major AETs at the BBB—which are transporters derived from both the SLC and ATP-binding cassette (ABC) gene families—and emphasize the differential expression of transporters in the multiple cells that comprise the neuro-vascular unit, including the capillary endothelium, capillary pericyte, the astrocyte endfeet or neuronal endings that contact the capillary basement membrane, and peri-vascular cells such as microglia [13]. There are seven ABC gene families, ABCA through ABCG, which encompass ~50 transporters. The most widely studied ABC transporters at the BBB are p-glycoprotein (ABCB1), breast cancer resistance protein, BCRP (ABCG2), and multidrug resistance protein MRP1-6 (ABCC1-C6) [13]. SLC transporters also contribute to the active efflux from the brain to blood of ligands and drugs, including members of the SLC21 family (now named the SLCO family), and include OATP1A2, and the mouse homologue, Oatp1a4 [13].

Nanoparticles comprise the sector in the field of brain drug delivery with the greatest number of publications [1]. Nanoparticles are a diverse group of formulations and include lipid nanoparticles (LNP)—which include cationic polyplexes (also called cationic liposomes)—and pegylated liposomes [1]. A review of the literature shows that nanoparticles, per se, do not cross the BBB, unless the nanoparticle is modified by conjugation of a Trojan horse ligand to its surface [1]. In this Special Issue, Thomsen et al. [14] describe the BBB transport of Trojan horse gold nanoparticles or Trojan horse LNPs, where the Trojan horse that mediates BBB transport is a TfRMAb. The BBB transport of Trojan horse LNPs in vivo is monitored using 2-photon microscopy [14].

Trojan horse LNPs are particularly suited to the BBB delivery of large nucleic acids such as mRNA or plasmid DNA. In this Special Issue, Sakurai et al. [15] describe the delivery of mRNA to cultured brain endothelial cells with LNPs, formulated without a Trojan horse, and encapsulating mRNA encoding for green fluorescent protein. The production of the LNPs described by the authors [15] is very similar to the production of the COVID-19 mRNA vaccines, which uses the ethanol dilution method for nucleic acid encapsulation within LNPs. The delivery of mRNA to cells with the pegylated liposome type of LNPs produced by Sakurai et al. [15] was only tested in cell culture, not in vivo, as these LNPs were formulated without a surface Trojan horse. However, when a TfRMAb or HIRMAb Trojan horse is conjugated to the surface of the LNP, the Trojan horse LNPs, also called Trojan horse liposomes (THL), enable the delivery of plasmid DNA to the brain in vivo [1]. Plasmid DNA encoding either reporter genes or therapeutic genes has been encapsulated in Trojan horse LNPs and administered IV in rats, mice, and monkeys [1]. THLs encapsulated with plasmid DNA encoding specific genes exert therapeutic effects in vivo in rodent models of brain cancer, PD, and Niemann-Pick type C1 disease [1].

BBB drug delivery is frequently measured in vitro with models of cultured endothelium, and these BBB models are discussed in detail in this Special Issue [1]. A critical examination of the in vitro models shows that they should supplement, not replace, in vivo measurements of BBB drug delivery. The in vitro BBB models are leaky owing to the marked down-regulation of BBB-specific gene expression when the brain endothelium is cultured in vitro [1]. The in vivo methods for the measurement of BBB drug delivery, compartmental model approaches, and the K_p,uu_ parameter are reviewed in this Special Issue by Bickel [16]. In a steady state, the K_p,uu_ parameter is the ratio of unbound drug concentration in brain interstitial fluid, relative to the unbound (bioavailable) drug concentration in plasma, which is equal to the ratio of the unidirectional clearance (CL) of influx, relative to the unidirectional clearance of efflux [16].

Continued progress in the field of brain drug delivery is important because of the rate-limiting role played by the BBB in the development of new drugs to treat diseases of the brain and spinal cord. Only ~2% of small molecules cross the BBB, and biologic drugs (recombinant proteins, RNA and DNA therapeutics) do not cross the BBB in the absence of a BBB delivery technology. Owing to the difficulty in the development of scalable BBB delivery technology that can be successfully translated to clinical medicine, the majority of brain drug delivery approaches either avoid the BBB (e.g., drug delivery to CSF, intra-cerebral drug delivery, trans-nasal drug delivery), or disrupt the BBB [1]. BBB disruption leads to the brain uptake of plasma proteins and to a sterile inflammatory reaction in the brain [1]. The alternative approach to brain drug delivery is to target the endogenous small and large-molecule transport pathways that normally function at the BBB. RMT pathways, which serve to deliver certain peptides to the brain (e.g., insulin or transferrin), can be targeted with molecular Trojan horses and IgG fusion proteins for the brain delivery of biologics. CMT pathways, which serve to deliver nutrients and vitamins to the brain, can be targeted for the brain delivery of small-molecule drugs. An understanding of the molecular and cellular biology of the endogenous transport pathways at the BBB is the key to the future development of brain drug delivery technologies.

## Data Availability

Not applicable.
